# An overview of mouse models of hepatocellular carcinoma

**DOI:** 10.1186/s13027-023-00524-9

**Published:** 2023-09-05

**Authors:** Hua-chuan Zheng, Hang Xue, Wen-Jing Yun

**Affiliations:** Department of Oncology and Central Laboratory, The Affiliated Hospital of Chengde Medical University, Chengde, 067000 China

**Keywords:** Animal model, Hepatocellular carcinoma, Immunotherapy

## Abstract

Hepatocellular carcinoma (HCC) has become a severe burden on global health due to its high morbidity and mortality rates. However, effective treatments for HCC are limited. The lack of suitable preclinical models may contribute to a major failure of drug development for HCC. Here, we overview several well-established mouse models of HCC, including genetically engineered mice, chemically-induced models, implantation models, and humanized mice. Immunotherapy studies of HCC have been a hot topic. Therefore, we will introduce the application of mouse models of HCC in immunotherapy. This is followed by a discussion of some other models of HCC-related liver diseases, including non-alcoholic fatty liver disease (NAFLD), hepatitis B and C virus infection, and liver fibrosis and cirrhosis. Together these provide researchers with a current overview of the mouse models of HCC and assist in the application of appropriate models for their research.

## Introduction

Liver cancer is the sixth most prevalent and the second deadliest malignancy in 2020 worldwide [1]. The foremost form among them is hepatocellular carcinoma (HCC), accounting for approximately 90% of cases, which has become a severe burden on global health [2]. Viral infections (e.g. hepatitis B or C) and chronic liver diseases (e.g. alcoholic liver disease and non-alcoholic fatty liver disease) are the main risk factors for HCC. Worldwide, HBV or HCV infection is the main cause of HCC, mainly in China, and most patients with HBV-related HCC experience cirrhosis [3, 4]. Non-alcoholic fatty liver disease (NAFLD) has gradually replaced viral hepatitis as the leading etiology of chronic liver disease in developed countries, of which 20% may develop into NASH and then HCC [5].

Increasing survival while maintaining the highest quality of life is the aim of HCC treatment. Resection, ablation, and transplantation are the mainstay of treatment for early-stage liver cancer. For advanced HCC, for many years only systemic therapy was used in HCC due to the resistance of HCC to cytotoxic chemotherapy [6]. To date, only a few drugs are available as first-line treatments, such as sorafenib and lenvatinib. Among second-line drugs, regorafenib is the only drug that can improve survival. Even as a first-line drug, Sorafenib only prolongs the median survival time of HCC patients by only 3–5 months, and the high incidence of primary and secondary drug resistance of sorafenib usually leads to treatment failure [7]. In recent years, dramatic advances in studying the relationship between tumor and tumor microenvironment, and the clinical successes of immune checkpoint blockade promote the development of cancer immunotherapy [8]. Multiple immune mechanisms such as checkpoint inhibition targeting PD-1, PD-L1, and CTLA-4 have been shown to be effective, tolerable, and clinically beneficial for advanced liver cancer. The combination of atezolizumab (anti-PD-1 antibody) and bevacizumab (anti-VEGF antibody) have been the best first-line treatment options for advanced HCC [9]. Moreover, the FDA also accelerated approval of the anti-PD-1 antibody pembrolizumab as monotherapy and a combination of nivolumab (another anti-PD-1 antibody) and ipilimumab (an anti-ctLA4 antibody) for second-line treatment of advanced liver cancer, based on efficacy data from early trials. However, these data have not been confirmed in a Phase III study. As of 2022, more than 20 phase III trials of ICI based combination therapies are currently underway [10]. However, only about 5% of new cancer drug candidates are approved by the Food and Drug Administration (FDA, USA) because the lack of preclinical models that accurately reproduce the human TME and immune system in the human liver has led to the failures of most preclinical trials [11]. Therefore, effective preclinical models are required to elucidate the etiology, carcinogenesis mechanism, and progression of HCC, and assess novel clinical therapeutic strategies, and there is a need to understand the determinants of response to and resistance to these different drugs and/or combinations in the patients with liver cancer.

Chronic infection with hepatitis virus and subsequently persistent immune response are major well-recognized risk factors for cirrhosis, and eventually HCC. The virus-associated hepatocellular carcinogenesis involves both viral and host factors. The host factors included a functionally inefficient CD8(+) T-cell response that fails to clear the infection but sustains a chronic necroinflammatory process [12]. Chronic immune-mediated liver cell injury triggers the development of HCC in the absence of viral transactivation, insertional mutagenesis, and genotoxic chemical [13]. Therefore, amelioration of immune-mediated chronic liver injury may prevent HCC by diminishing intrahepatic HBV-specific CD8(+) T cells and HBV-nonspecific inflammatory cells. Nakamoto et al. [14] demonstrated that neutralization of the activity of Fas ligand prevented hepatocyte apoptosis, proliferation, liver inflammation, and the eventual development of HCC using a unique animal model of chronic hepatitis that induced hepatocellular carcinogenesis. However, Zong et al. [15] found that expression of TIGIT, a promising immune checkpoint in tumor immunotherapy, increased with age on hepatic CD8 + T cells in HBsAg-transgenic (HBs-tg) mice whose adaptive immune system was tolerant to HBsAg, while TIGIT blockade caused chronic hepatitis and fibrosis, along with the emergence of functional HBsAg-specific cytotoxic T lymphocytes (CTLs). In chronic inflammation and fibrosis of NAFLD, Shalapour et al. [16] observed accumulation of liver-resident immunoglobulin-A-producing (IgA+) cells, which expressed PD-L1 and interleukin-10, and directly suppress liver cytotoxic CD8 + T lymphocytes, which prevent hepatocellular carcinogenesis and express a limited repertoire of T-cell receptors against tumour-associated antigens.

Experiments that are difficult or impossible to perform in humans can be carried out in mice [17]. Many existing mouse models of HCC are available, such as genetically engineered mouse models (GEMMs), chemically-induced models, and implantation models. Since cancer immunotherapy, a new therapy that uses the human immune system to attack cancer, has made breakthroughs in cancer treatment, some humanized mice that may mimic the human immune response have been developed [18, 19]. For researchers, appropriate mouse models should be selected according to their study. The review provides an overview of the classical mouse models for HCC and their application in studying the immunotherapy of HCC.

## Virus-related mouse models

Chronic infection with HBV or HCV is the most serious form of viral hepatitis due to more severe manifestations of an accelerated progression to liver fibrosis, cirrhosis, and HCC. HBV exhibits a narrow species tropism and robustly infects humans and higher primates, such as chimpanzees, gorillas, gibbons, and orangutans due to the lack of HBV receptors on other animal hepatocytes, like mouse. Although surrogates allow the infection of HBV, their host genetic backgrounds, immune responses, and molecular virology differ from those of HBV. To promote in vivo HBV research, and evaluate therapeutic effects of chronic hepatitis B, it is essential to understand the barriers towards interspecies transmission and develop human chimeric mice [20, 21].

To bypass this entry step of HBV infection, Huang et al. [22] developed a novel HBV model in immunocompetent mice by hepatic delivery of the HBV genome using trans-splicing adeno-associated viral vectors (AAV/HBV). Importantly, 12–16 months later, all 12 AAV/HBV-transduced mice developed macroscopically visible liver-tumor nodules, and characterized with typical HCC features. Wu et al. [23] generated a mouse model of spontaneous HBV-related HCC by replacing wild-type hepatocytes with HBsAg + hepatocytes (namely HBs-HepR mice). The tumors in HBs-HepR mice were inflammation-associated HCC, characterized by increased CD8 + T cells and their low production of IL-2, TNF-α, and IFN-γ, and similar to HBV-related HCC in patients, which was distinguished from diethylnitrosamine-induced HCC, TGF-β-activated kinase 1 knockout-induced HCC, HCC in a stelic animal model, or NASH-induced HCC. Hao et al. [24] used immunocompetent Fah-/- mice as the recipients to establish HBs-HepR mice, which exhibited persistent HBsAg expression and CD8 + T cells infiltration with chronic hepatitis and eventually developed HCC. Nakamoto et al. [25] have developed a transgenic mouse model of chronic immune-mediated liver disease that induced hepatocellular carcinogenesis. HBsAg-specific CTLs were detected as hepatic CD8 + T lymphocytes, and that monocytes/macrophages were significantly increased as the disease developed.

Chung et al. [26] generated transgenic mice expressing HBV polymerase (HBp) or the RT domain of HBp, which developed early cirrhosis with steatosis by 18 months and 10% developed HCC because HBp stimulated coordinated proapoptotic and proinflammatory responses. Kim et al. [27] have established transgenic mice harboring entire HBx gene under its own regulatory elements, which displayed multifocal areas of altered hepatocytes, followed by benign adenomas and HCC. Male mice developed disease and died much earlier than females.

Besides humans, HCV infection can be experimentally transmitted to chimpanzees because they have related innate and adaptive immune responses. However, limited availability, high cost and ethical considerations limit their application. The only small animals of robust HCV infection are highly immunodeficient mice with human chimeric livers, but they cannot be employed to investigate adaptive immune responses. Novel strains of immunodeficient mice have been developed that allow for the engraftment of human hepatopoietic stem cells, as well as functional human lymphoid cells and tissues, effectively creating human immune systems in otherwise immunodeficient mice. Additionally, transgenic mice should be developed to clarify the pathogenesis of HCV-related HCC [28]. Labonté et al. [29] orthotopically implanted HCC cells in athymic nude mice, and found a close correlation between HCV RNA level and tumor size and the immunoreactivity to HCV-encoded NS5B protein in tumor cells.

Using transgenic mouse models, the core protein of HCV was found to have an oncogenic potential although continuous inflammation or environmental factors were also involved in hepatocarcinogenesis. The pathways for the oncogenic roles of HCV core protein included the augmented oxidative stress without inflammation and the aberrant cellular gene expression and intracellular signaling transduction. Alcohol feeding further activated the two pathways synergistically with HCV, resulting in hepatocellular carcinogenesis [30]. The patients persistently infected with RNA HCV had chronic inflammation resulting from immune responses against infected hepatocytes, which was associated with progressive fibrosis and cirrhosis, and then HCC [31]. Islam et al. [32] generated transgenic mice expressing the genome RNA of HCV in the hepatocyte produced ~ 3 × 10^6^ HCV RNA copies/mL serum and showed hepatic steatosis without any necroinflammation at the age of 6 months or hepatocellular carcinoma at the age of 15 months. Transgenic mice were established with tetracycline-inducible coexpression of HCV core or HCV open reading frame and luciferase. The histology of liver sections provided evidence of steatosis, which was correlated with an inflammatory response [33]. Pasquinelli et al. [34] produced transgenic mice that expressed the HCV core protein in the liver under the transcriptional control of the mouse major urinary protein promoter, but didn’t find histological or biochemical evidence of liver disease or HCC.

Viral hepatitis and aflatoxin B1 (AFB1) exposure are common risk factors for HCC. Ueda et al. [35] exposed AFB1 to HBx transgenic mice and found that AFB1 acted synergistically with HBV to accelerate the development of HCC. In contrast, no p53 mutations were found in HCC. Jeannot et al. [36] also observed hepatic adenomas or carcinomas and preneoplastic lesions (hyperplasia or foci) in 22.5% (9 of 40) of AFB1(6 µg/g bw)-treated WT mice. In AFB1-treated HCV-Tg mice, the incidence of tumorous or pretumorous lesions was significantly elevated (50%, 18 of 36), with the difference largely due to a 2.5-fold increase in the incidence of adenomas (30.5 vs. 12.5%). Here, we summarize all the virus-related mouse models in Table 1.

**Table 1:** The virus-related mouse models

Author	Mouse model type	Mouse strains	Method
Huang et al. [22]	Live tumors	BALB/c, C57BL/6, FVB and ICR	AAV/5′-HBV-SD、AAV/3′-HBV-SA, AAV/5-HBV-SD, AAV/3-HBV-SA (AAV/HBV) were intravenously injected into mice at 6–8 weeks. Macroscopic liver tumors appeared between 12 and 16 months after AAV/HBV trans induction.
Wu et al. [23]	HCC	HBs-Tg, (Fah−/−), C57BL6/J,	Liver parenchymal cells from HBS TG mice were transfused into NTBC-treated FAH deficient mice with healthy immune system at 8–12 weeks of age via spleen injection. HBs-HepR mice with liver replacement reconstruction exhibited chronic hepatitis, liver fibrosis, and liver cancer.
Hao et al. [24]	HBs-HepR mice	C57BL/6J	Hepatocytes from HBS-TG mice were transfused into NTBC-treated FAH deficient mice with healthy immune system at 8–10 weeks of age via spleen injection. HBs-HepR mice with liver replacement reconstruction exhibited HCC at 9 months after reconstruction.
Chung et al. [26]	TG mice expressing HBp or the RT domain of HBp	C57BL/6 N	The 5.3-kb DNA containing AAP-HBp or the 3.9-kb DNA containing AAP-RT was microinjected into the pronuclei of fertilized mouse eggs.
Kim et al. [27]	Transgenic mice harboring entire HBx gene	CD1	The HBx transgenic mice were derived by microinjection of a 1.15 kb HBV subtype adr DNA fragment into single-cell embryos of CD1 mice.
Labonté P et al. [29]	Transplantation model of HCC	CD1	HuT7-3 cells were injected directly into the liver of nude mouse.
Islam et al. [32]	TG mice expressing HCV RNA	C57BL/6	HCV JFH-1 DNA was injected into a male pronucleus of fertilized eggs.
Ernst et al. [33]	HCV TG mouse	C57BL/6x	HCV core-transgenic mice were obtained by pronuclear injection of a purified 4.9 kb AsnI–XmnI fragment of pBI-L core containing the bidirectional expression unit into F1-zygotes (C57Bl/6 X DBA (H2b)). Similarly, the HCV ORF mice were generated by transferring the 13.5 kb fragment of pBI-L ORF into fertilized eggs.
Jeannot et al. [36]	Chemically-induced mouse models	C57BL/6	Neonatal mice were administered a single dose of Aflatoxin B1 (6 µg/g body weight) or tricaprylin vehicle (15 µl/g body weight) by intraperitoneal injection.

Note: HCC, hepatocellular carcinoma; TG, transgene; HBV, hepatitis B virus; HCV, hepatitis C virus; HBp, HBV polymerase

## Genetically engineered mouse models

**Fig. 1:**
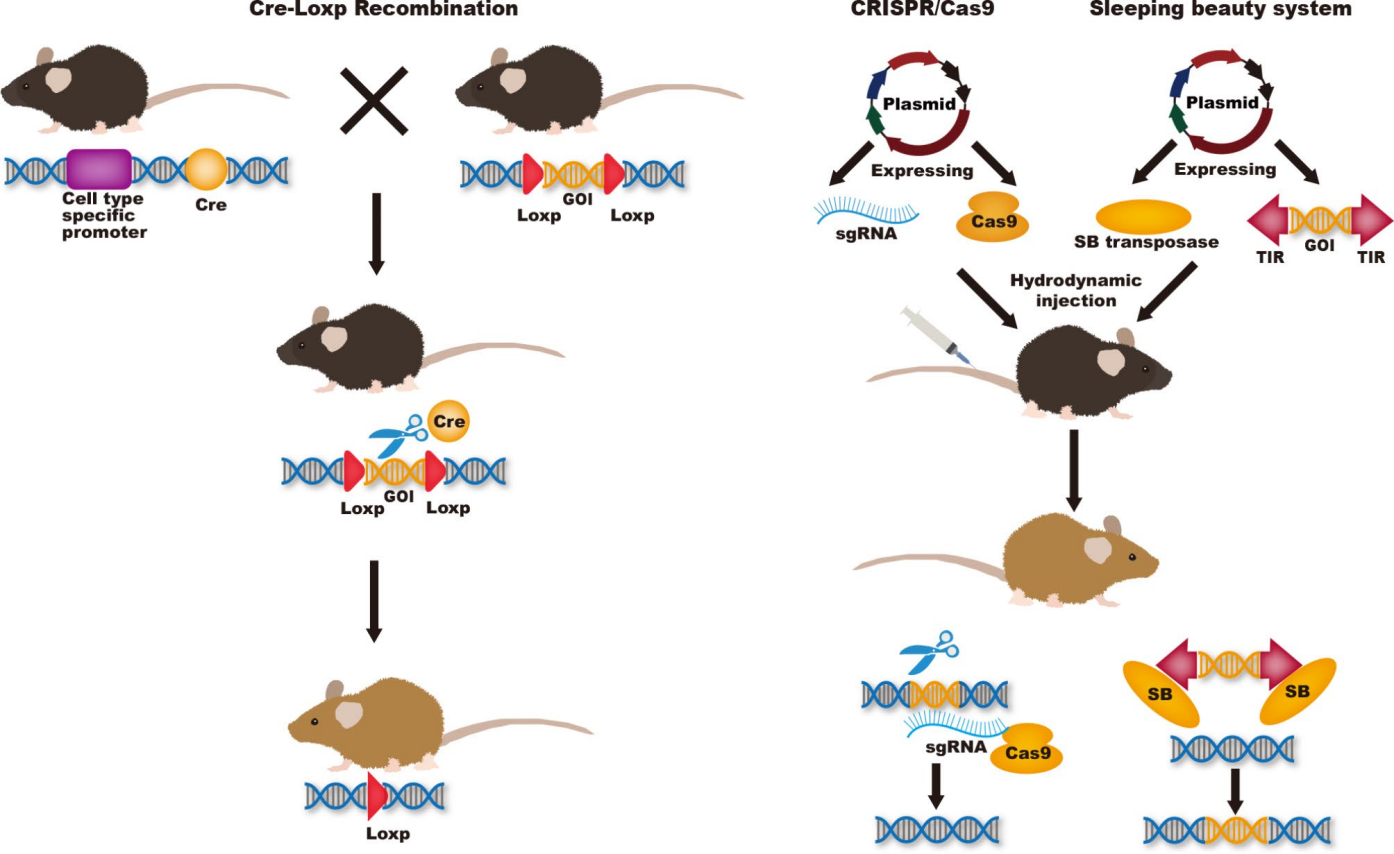
Commonly used techniques to establish GEMMs including Cre-Loxp recombination, CRISPR-Cas9, and Sleeping Beauty transposase system

The tumorigenesis and progression of HCC is a complex, multi-step, multi-factorial, and multi-gene-involved process. Although molecular mechanisms of hepatocarcinogenesis remain unclear, many of the genetic and epigenetic alterations that contribute to HCC were considered to be associated with increased reactive oxygen species, inflammatory cytokines, and fibrosis [37, 38]. GEMMs are a powerful tool to further investigate the molecular mechanisms of HCC development. Such models can also be used to identify the genes involved in HCC development by overexpressing or knocking out these genes in the liver to observe the biological, pathophysiological, and functional changes. The GEMM model can also be used to study tumor response to immunotherapy in the tumor immune microenvironment. Commonly used techniques to establish GEMMs include Cre-Loxp recombination, CRISPR-Cas9, and Sleeping Beauty transposon system. (Fig. 1)

Genetic mutants in GEMMs models can be manipulated in germ or be induced in adult mice or in a tissue-specific way. Genes targeting in ES cells knockout harbor a null allele in their germline. This model provides appropriate way to study the function of the gene of interest in systemic physiology or pathology. Although the construction time of this model is short and the mating process of mice is simple, one third of mice will die in the embryo after gene knockout and may lead to compensation of other genes, resulting in no significant phenotypic changes after knockout. Furthermore, knocking out a gene simultaneously in an entire organism can produce complex results, with too many interfering factors to understand the gene’s specific function. Accurate models of these diseases must achieve tissue and/or stage-specific control of these genetic mutations. Thus, various conditional gene knockout technologies were created and developed [39]. Cre-Loxp system is extensively used as a powerful tool to generate conditional gene knock-out mice. Cre recombinase is placed in downstream of a specific promoter, such as albumin in the liver. Gene can be catalyzed by Cre recombinase by adding two loxP sites to both sides of the target gene. To obtain stage-specific control of these genetic mutations, tet-on/off system and tamoxifen system were developed. The activation of Cre recombinase is manipulated by tetracycline or tamoxifen to achieve genetic tissue and stage-specific mutations [39]. Conditional knockout mouse models can be used to study embryonic lethal genes, as well as to objectively and systematically study the role and mechanism of target genes in different tissue organogenesis, development, disease occurrence and treatment. The disadvantages of this model are the relatively long cycle and the complex mating process of mice. In addition, Human tumors are usually formed by the accumulation of mutations in a small number of cells and finally transformation. However, the conditional knockout model deletes target genes in all cells expressing promoter, resulting in tumor mutations in the model may not be able to fully mimic human diseases [40, 41]. Hydrodynamic injection allows specific expression of transgenic plasmid in the liver, which is a flexible, time and cost-saving technique [42]. Xue and colleagues investigated the potential of using CRISPR-Cas9 system to create somatic mutations in adult mice. They delivered a pX330 vector9 co-expressing an sgRNA targeting PTEN and Cas9 to the liver by hydrodynamic injection. CRISPR-mediated mutations in PTEN had the same effects as gene deletion using Cre-LoxP, both resulting in increased phosphorylation of Akt and lipid accumulation in hepatocytes. They then used the same method to achieve simultaneous silencing of pten and p53 genes in liver cells and successfully induced liver tumors that mimicked those caused by Cre-loxP mediated deletion of Pten and p53 in 3 months, supporting the use of multiplexed CRISPR editing of cancer genes in liver [43]. Bell and colleagues described a procedure for efficient delivery of the SB transposon system to the liver of mice using hydrodynamic injection. One day after successful injection, 5–40% of hepatocytes expressed the target gene. Thereafter, transgene expression remains stable at 1% of the level at 24 h, demonstrating transposon integration into the chromosome [44]. Hydrodynamic injection does not require modifying ES cells, nor does it require breeding multiple mutant animals to produce complex mutations. This approach allows faster testing of any single gene or combination of genes suspected of being able to initiate tumor formation in the liver [43].

Advances in sequencing technology have made it possible to detect liver cancer genomes at high resolution. DNA sequencing and mutation analysis revealed high-frequency mutations of multiple liver cancer genes, including up-regulated genes such as MET and MYC and down-regulated genes such as PTEN, RB1, TP53 and CTNNB1, and multiple signal pathways, including p53 pathway, WNT pathway, NF-κB pathway, and TGF-β signal pathway and so on [45, 46]. The role of these liver cancer driver genes in the development of liver cancer can be further understood through GEMM models. The AlfpCre + Trp53^Δ2–10/Δ2–10^ mice were induced liver tumor in 14 to 20-month-old mice by Cre-LoxP recombination [47]. Tumors were detected in mouse liver 3 months after simultaneous knockout of PTEN and p53 gene by hydrodynamic transfection with Crispr-Cas9 system, while no tumors were detected 4 months after PTEN knockout alone [42]. In a study investigating the role of MYC and E2F1 genes in the development of liver cancer, 22% of 6-month-old and 100% of 12-month-old c-Myc/E2F1 double transgenic mice were induced to develop liver tumors, while only 23% of Alb/c-Myc and 60% of Alb/E2F1 mice were induced to tumors, and no tumors were induced in c-Myc or E2F1 deletion alone mice [48]. Likewise, Tward and colleagues overexpressed MET and deleted CTNNB1 in mice by hydrodynamic transfection with transposable vectors. Liver tumors were detected in 74% of 1-month-old mice, while no tumors were detected in MET overexpression alone or CTNNB1 deletion alone mice [49]. Cao and his colleagues used the CRISPR-Cas9 and Sleeping Beauty systems to construct mutants that were delivered by hydrodynamic injection into mouse livers, where PTEN and p53 were knocked out and MET and CTNNB1 were overexpressed. Mice with this multi-gene combination mutation developed liver cancer in just six weeks [50]. These results indicate that the tumor induction time by multi-gene mutation is shorter and the tumor formation rate is higher than that by single gene mutation. The hepatocellular carcinogenesis is the result of the accumulation of multiple oncogenic driver gene mutations, and the realization of multi-oncogenic mutation technology can provide better models for the study to human HCC.

Ochiai et al. [51] observed spontaneous HCC in hepatic TFF1-deficient mouse model with a higher nuclear-localized β-catenin expression. Visible premalignant liver tumor nodules were found in miR-122a^−/−^; PTEN^+/−^ and miR122a^−/−^; Alb-cre; PTEN ^f/+^ mice at 6 months of age, and closely linked to inflammatory microenvironments [52]. Hepatic knockout of Tsc1, PTEN, and both genes developed liver tumors, and the onset of liver tumors in Tsc1^f/f^; Alb-cre mice was later than in the other strains and these were predominantly HCC. There also appeared cholangiocarcinomas in PTEN ^f/f^; Alb-cre mice.

The tumors in Tsc1^f/f^; PTEN ^f/f^; Alb-cre mice were larger than other strains and histologically had mixed architectures [53]. Reportedly, Alb-cre; Ctnnb1^f/f^ mice had efficient deletions of β-catenin in hepatocytes at age of 2 months, but β-catenin-positive hepatocytes reappeared with aging. In 12-month-old mice, β-catenin–expressing hepatocytes existed in pericentral area, but not in periportal one. β-catenin–positive hepatocellular adenomas and carcinomas were mostly observed in 1-year-old mice. In Alb-cre/T antigen mice, we found HCC and peritoneal spreading as evidenced by ascites, CT scanning, HE and immunohistochemical staining [54]. We also found primary HCC, and metastatic cancers in the spleen, lung and peritoneum, which showed strong T antigen expression [55]. The histologically-normal oncogenic hepatocytes from young male SV40 T antigen (TAg)-transgenic mice were intra-splenically injected into the immunocompetent male C57BL/6J mice, which suffered from liver fibrosis by induction of a carbon tetrachloride. T antigen was expressed under control of a liver-specific promoter by ndrogen from recipient male mice at puberty and thereby transferred hepatocytes of cirrhosis into cancer cells, which mimics human HCC initiation and progression in liver fibrosis/cirrhosis [56]. The oncogene activation and tumor suppressor inactivation in hepatocytes can develop liver tumor, especially HCC, which are helpful to investigate the role of genetic alteration in hepatocellular carcinogenesis.

The clinical efficacy of PD-1 pathway inhibition as a monotherapy is limited to most patient subpopulations of the tumor types studied, with response rates of 20% or less in many cancers [57]. Galarreta and colleagues created a novel GEMM model of HCC. They conducted antigen-free MYC-LUC and MYC-LUCOS with exogenous antigens transposon-base vector, and sg-p53 CRISPR-Cas9 vector. Hydrodynamic tail vein injections was used to deliver DNA specifically into the hepatocytes to create MYC-LUC; sg-p53 mice and MYC-LUCOS; sg-p53 mice. Wild-type mice with MYC-LUCOS; sg-p53 had a longer survival time than the Rag^−/−^ mice, confirmed that the lymphocytes eliminated foreign antigens. Further experiments proved that CD8 + T cells played a major role. They then performed GSEA analysis and RNA sequencing, confirming that β-catenin was activated during immune escape, and β-catenin can promote immune escape by impairing dendritic cell recruitment in the context of HCC, and immune surveillance can be restored by CCL5. They finally found that β-catenin-driven tumors were resistant to anti-PD-1 [58]. Galarreta et al. ‘s study provides researchers with a complete idea of using GEMM model to study the mechanism of PD-1 resistance, and created a novel GEMM model for the study of liver cancer that interrogates how different genetic alterations affect immune surveillance and response to immunotherapies [58].

Genetically-engineered mouse models enable investigators to study the effects of specific gene mutations on tumorigenesis or detect new targets of gene therapy by activating oncogenes or inactivating tumor suppressor genes [47]. Here, we summarize all the GEMM in Table 2.

**Table 2:** The genetically engineered mouse models of hepatocellular carcinoma

Author	Model	Technology	Mouse strains	Method
Xue et al. [43]	HCC	Sleeping beauty CRISPR-Cas9 system	FVB	Hydrodynamic injection of a CRISPR plasmid DNA expressing Cas9 and sgRNAs to the liver and directly target the tumor suppressor genes Pten and p53, alone and in combination.
Bell et al. [44]	HCC	Sleeping beauty transposon system	C57BL/6	The rapid, high-pressure injection of a naked plasmid DNA solution (2ml DNA/20 g mouse) into the tail vein within a period of 4 − 7 s.
Katz et al. [47]	HCC	Cre-loxp	C57BL/6	Conditional Trp53^F2–10/F2–10^ KO mice were crossed with AFP-cre mice.
Calvisi et al. [48]	HCC	Cre-loxp	C57	TG mice were generated by crossing homozygous c-Myc with homozygous E2F1 mice.
Tward et al. [49]	HCC	Sleeping beauty transposon system	FVB/N	Ten to 50 micrograms of the plasmids encoding the Sleeping Beauty transposase and transposons with oncogenes of interest in a ratio of 1:25 were diluted in 2.5 ml of filtered 0.9% NaCl and then injected into the lateral tail veins of 6- to 8-week-old mice.
Cao et al. [50]	HCC	CRISPR-Cas9 and sleeping beauty transposon system	C57BL/6	Plasmids pCMV/SB, PT3-EF1a-c-Met, PT3-△90-β-catenin, Lenti CRISPR-sgPten and LentiCRISPR-sgp53 were injected into mice at a dosage of 10 µg/mouse within 3–5 s/time.
Ochiai et al. [51]	HCC	Cre-loxp	C57BL/6	Conditional TFF1 KO mice were crossed with Alb-cre mice to generate a TFF1-deficient HCC mouse model (KC/TFF1−/−).
Kenerson et al. [53]	HCC	Cre-loxp	C57BL/6	Tsc1^f/f^ and Pten^f/f^ mice were separately bred with Alb-cre mice to generate Tsc1^f/f^; Alb-cre and Pten^f/f^; Alb-cre mice respectively. Double knockout mutant mice (Tsc1^f/f^; Pten^f/f^;Alb-cre) were created by crossing Tsc1^f/f^;Alb-cre with Pten^f/f^; Alb-cre mice to generate Tsc1^f/+^;Pten^f/+^;Alb-cre mice.
Sekine et al. [54]	HCC	Cre-loxp	C57	Conditional Ctnnb1 KO mice were crossed with Alb-cre mice to generate an Alb-cre; Ctnnb1^f/f^ mouse model, which developed hepatocellular adenomas and carcinomas at age of 1 year.
Zheng et al. [55]	HCC	Cre-loxp	C57BL/6	We crossed CAG-loxp-LacZ T antigen mice with Alb-cre mice, and observed HCC at 3–10 months of age.
Ruiz et al. [58]	HCC	CRISPR-Cas9 system	C57BL/6	Hydrodynamic injection of px330-sg-p53 and CMV-SB13 in combination with MYC-luc or MYC-lucOS into 6-week-old female mice. The majority of MYC-luc; sg-p53 mice presented gross liver tumors that caused death with a median survival of 35–44 days.

Note: HCC, hepatocellular carcinoma; TG, transgene; KO, knockout

## Chemically-induced mouse models

The liver is always exposed to hepatotoxic compounds that affect liver homeostasis and induce cancer [59]. For several decades, chemotoxic agents have been widely utilized to induce tumor formation in mice to reproduce human diseases, study the pathogenesis of the diseases, and assess candidate therapeutics. Genotoxic carcinogens can directly cause tumorigenesis by damaging DNA structure, while most carcinogenic agents, namely non-genotoxic (or epigenetic) carcinogens, lack the ability for initiation and promotion, and mainly indirectly promote tumor formation by converting cells from the promotion stage to the progression stage [60, 61].

DEN, a genotoxic carcinogen, is widely used to induce HCC in rodents since 1966 [62]. DEN alkylates DNA to form DNA adducts resulting in hepatocarcinogenesis [63]. Four mutated genes are identified as putative oncogenic drivers in DEN-induced HCC, including *Braf, Hras, Egfr*, and *Apc* [64]. However, mutations in these genes are rarely found in human liver cancer, and the telomere maintenance, WNT/b-catenin signaling, and P53 cell cycle control are the most common cellular processes and pathways in the pathogenesis of human HCC [64]. The time for HCC formation by a single DEN administered is not only dose-dependent, but also related to other factors including age, sex, and genetic background [65, 66]. Compared with adult mice, infant mice show a higher induced rate of HCC, which might be attributed to a faster replication of hepatocytes in infant mice. Under a low dose of nontoxic concentration (0.312 to 5.0 ug/g body weight) of DEN, infant male B6C3F_1_ mice were successfully induced to develop HCC, while a higher DEN dose of up to 50 ug/g failed to induce any nodular lesion in young adult mice (42 days of age) [67]. In addition, HCC was more easily induced in male mice associated with inflammation. This may be because inflammatory cytokines, chemokines, and tumor-related leukocytes and platelets candirectly participate in the development of malignant tumors [68]. When exposed to DEN, serum IL-6 was observed lower in female mice than in males because estrogen can reduce IL6 concentrations. IL-6 and TNF participated formation of obesity-promoted HCC by causing hepatic inflammation and activation of STAT3 [69]. Therefore, some researchers proposed that estrogen could be administrated to prevent male liver cancer [70]. For adult mice failing to induce HCC by administering a single DEN, other tumor promoters such as carbon tetrachloride (CCl4), phenobarbital, and high-fat diet feeding, were required [69, 71–73]. CCl_4_ can induce cell damage by attacking hepatocytes by free radicals generated by the cytochrome P-450-dependent step. After liver exposure to CCl_4_, early damage is the result of a direct effect of the CCl_4_ toxicity, this damage is partially reversible, and subsequent damage may be mediated by lipid peroxidation [74]. After a single administration of DEN (Dissolve 1 mg DEN in 15 mL PBS on the day of dosing) in 2-week-old mice, CCl_4_ (Dissolve 1 ml CCl4 in olive oil at the final volume of 10 ml) was repeatedly administered for 14 weeks starting at week 8. All mice developed HCC tumors within 5 months. Moreover, this model can be used to study fibrosis- and inflammation-related HCC [72, 75]. The development of human HCC generally undergoes the process of liver fibrosis to cirrhosis and then cancer. The combined induction of DEN and CCl4 not only improves the shortcomings of the longtime taken by traditional chemical induction, but also better simulates the development process of human liver cancer. Interestingly, when given a DEN followed by phenobarbital treatment, phenobarbital can enhance DEN’s ability to induce HCC, but the opposite effect occurs when phenobarbital is used concurrently with DEN [76].

Dietary exposure to aflatoxin B1 (AFB1) is also thought to be a high-risk factor for HCC [77]. AFB1-induced HCC models are most performed on rats, and many mouse strains are resistant to AFB1. Seven-day-old C57BL/6J and DBA/2J mice were injected with 6 µg/g body weight of AFB1. By 52 weeks of age, HCC was detected in 90% of DBA/2J male mice while only 27% of C57BL/6J male mice developed HCC. Analyzing the genetic differences between the two mice may be a way to help us find targets against aflatoxin-induced HCC. 100% of hepatitis B surface antigen (HBsAg)-transgenic mice (C57BL/6J-transgene [Tg]N [Alb1HBV]44Br) developed HCC, and AFB1 exposure accelerated HBsAg-induced hepatocellular carcinogenesis [70]. The results remind us that preventing exposure to aflatoxin in HBV-infected populations is necessary.

Chemically-induced models can mimic to some extent the progression of HCC, including injury, cirrhosis, and finally tumor, but seems in a more artificial process that does not reflect the real condition. Moreover, the long time required to induce liver cancer by chemically-induced method, the high mortality rate, and the uneven occurrence time, location and number of lesions of liver cancer between individuals also limit the use of this method. Here, we summarize all the chemically-induced mouse models in Table 3.

**Table 3:** The mouse models of chemically-induced hepatocellular carcinomas

Author	Mouse Model Type	Mouse strains	Method
Vesselinovitch et al. [67]	Chemically-induced mouse models	C57BL/6J and C3HeB/FeJ F_1_	C57BL/6J female mice was bred with C3HeB/FeJ F1 male mice to generate C57BL/6J × C3HeB/FeJ F1 mice model(B6C3F1). A single intraperitoneal injection of DEN was administered to 15-day-old B6C3F1 male mice. The B6C3F1 mice were divided into two groups. The first groups of mice were given 0.625, 1.25, 2.5, and 5.0 µg of DEN per g of body weight. Another group of mice was given 0.312, 0.625, 1.25, 2.5, and 5.0 µg of DEN per g of body weight. Under a low dose of nontoxic concentration (0.312 to 5.0 ug/g body weight) of DEN, infant male B6C3F1 mice were successfully induced to develop HCC after an average of 44 weeks.
Leenders et al. [70]	Chemically-induced mouse models	C57BL/6J and DBA/2J	Seven-day-old mice were injected with 6 µg/g body weight of AFB1. By 52 weeks of age, HCC was detected in 90% of DBA/2J male mice while only 27% of C57BL/6J male mice developed HCC.
Uehara T et al. [75]	Chemically-induced mouse models	B6C3F_1_	Male mice are administered a single intraperitoneal injection of 1 mg/kg DEN (Dissolve 1 mg DEN in 15 mL PBS on the day of dosing) at 14 days of age. Beginning at 8 weeks of age the animals are intraperitoneally administered 0.2 ml/kg CCl_4_ (Dissolve 1 ml CCl_4_ in olive oil at the final volume of 10 ml) two times per week for up to 14 weeks.

Note: DEN, diethylnitrosamine; AFB1, Aflatoxin B1

## Implantation models

Implantation models are the most common methods to establish HCC tumors by subcutaneously or orthotopically injecting HCC cell lines or tumor tissue fragments into mice [78]. These models are easy to conduct and yield reproducible data, making them widely used to test new anti-tumor agents and therapeutic strategies.

According to whether the tumor was transplanted to its original tissue, implantation models are divided into orthotopic and heterotopic models. Heterotopic models, in which cells or tumor tissues are usually injected subcutaneously, have the advantage of allowing direct observation of tumor growth and its response to anti-tumor agents. Compared with heterotopic models, orthotopic models are more commonly used in HCC than in other cancer fields because the liver is not only a metabolic organ, but also a key immune tissue, and its tumor microenvironment is very complex; therefore, orthotopic models can better mimic the tumor microenvironment of HCC [79, 80]. However, orthotopic implantation needs more challenging technologies, another important point to consider is that the microenvironment of the mouse liver is also different from that of humans.

The main difference between syngeneic and xenograft models lies in the different HCC cell lines and tumor tissue sources. Xenograft models (usually patient-derived xenograft models, PDX) using patient HCC cell lines or tumor tissue require to be performed in immunodeficient mice to avoid rejection and therefore cannot be adequate for tumor immunotherapy studies. Athymic nude (Foxn1^nu^) mouse, a mouse strain deficient in T lymphocytes and characterized by hairlessness, is widely used in this approach to avoid the immune system’s rejection of foreign tissues. But their other immune response is normal except for T-dependent antigens [81, 82]. A severe combined immunodeficiency (SCID) mouse was established and characterized by a lack of both functional T and B lymphocytes [83, 84]. However, the gradual increase of T and B lymphocytes as age and the existence of NK cells limit the use of this strain [85]. The non-obese-diabetic severe combined immunodeficient (NOD-SCID) mice is another common-used mouse strain. Distinguished with the high incidence of autoimmune and insulin-dependent diabetes mellitus of non-obese-diabetic mice (NOD) mice, NOD-SCID mice are both insulitis- and diabetes-free, and lack T and B lymphocytes, and have low NK cell activity. This more radical immunodeficiency of both innate and adaptive immunity provides a better environment for reconstitution with human hematopoietic cells. But the high incidence of thymic lymphomas, the shorter lifespan of a mean of 8.5 months, and the high sensitivity of irradiation limit the utility of this strain [86, 87]. Based on NOD-SCID mice, NOD/LtSz-scid interleukin-2 receptor common gamma chain deficient (IL2rγ^null^) (NSG) and NOD/Shi-scid IL2rγ^null^(NOG) were established. The differences between them are mainly genetic backgrounds and IL2Rγ exon deletion that which NOG is exon 7 deletion while NSG is exon 1. The two strains have a higher engraftment level, a lower NK activity, a relatively low irradiation sensitivity, and a longer lifespan of about one and a half years [88–90]. The BALB/c Rag2^null^ IL2rγ^null^ mouse is a non-commercial immunodeficiency mouse that is less sensitive to irradiation than NOG/NSG mice because of their innate resistance to X-rays [90, 91]. Recently a new gamma‑radiated immunosuppressed (GIS) tumor xenograft mice was established as a new human in vivo xenograft tumors model, which can survive in an unclean animal room and have the potential to be applied to a wide range of biomedical cancer studies [92]. Although allograft mice have a mature immune system, implanted tissues or cells are derived from the mice and cannot adequately reflect the characteristics of human HCC. Despite mice sharing similar genes to humans, fundamental phenotypic and functional differences exist between the immune systems of humans and mice [18, 93].

In HepG2-derived xenograft model and a PDX model of HCC, CDK9 inhibitor, PHA767491 and oroxylin A (OA) from Scutellaria baicalensis significantly decreased the protein expression of CDK9, PINK1, PRKN, p-SIRT1, FOXO3 and BNIP3 in tumor tissues. In HepG2-derived xenograft model, the combination of OA and sorafenib had stronger tumor growth delay activity than either monotherapy, and the increase in tumor weights was significantly inhibited by this combination therapy. Sorafenib significantly upregulated the protein levels of PINK1 and PRKN in tumor tissues, while OA strongly reduced these levels. These findings demonstrated that OA could delay tumor growth and improve the therapeutic effects of sorafenib by inhibiting PINK1-PRKN-mediated mitophagy [94]. Su et al. [95] detected a higher level of m6 A reader YTH N6-methyladenosine RNA binding protein 1–3 (YTHDF1) in the sublethal-heat-exposed transitional zone close to the ablation center than that in the farther area using an IRFA (insufficient radiofrequency ablation) HCC orthotopic mouse model. Both m6 A modification and YTHDF1 protein level were elevated in HCC PDX mouse model. YTHDF1 knockdown drastically restrains the tumor metastasis evoked by sublethal heat treatment in tail vein injection lung metastasis and orthotopic HCC mouse models. Sublethal heat treatment enhanced epidermal factor growth receptor (EGFR) m6 A modification and promoted its binding with YTHDF1 to facilitate the translation of EGFR mRNA in the IRFA HCC PDX mouse model. Xun et al. [96] found that AST-3424, a novel specific aldo-keto reductase 1C3 (AKR1C3) prodrug, released a DNA alkylating reagent upon reduction by AKR1C3, and could inhibit tumor growth in HCC PDX models and orthotopic models. Hu et al. [97] observed the significantly synergistic anti-tumor effects after treatments with oncolytic adenovirus expressing Hsp70 combined with intravenously infusion of the cytokine-induced killer (CIK) cells into the PDTX model mice of HCC because adenovirus-mediated Hsp70 expression allowed the CIK chemotaxis in cancer tissues, and induce the infiltration of CD3 + T cells in tumor stroma. Using immunodeficient mice and HCV-related HCC tissues, Nazzal et al. [98] developed HCC-PDX model, similar to the patient primary tumor at the histological appearance and c-Kit expression. c-Kit inhibitor imatinib significantly reduced HCC-PDX xenograft tumor growth and phospho-Akt and cyclin D1 expression. Here, we summarize all the implantation models in Table 4.

**Table 4:** The implantation mouse models of hepatocellular carcinomas

First Author	Mouse Model Type	Mouse strains	Method
McClendon et al. [81]	HCC	FVB	Alb-cre mice were crossed to conditional Rb and/or p53 KO mice to generate a model for tissue-specific inactivation of Rb and p53. 14-day old mice were given a single interperitoneal injection (20 mg/kg) of DEN.
Duchosal et al.[86]	The hu-PBL-SCID mouse model	SCID	6 week-old SCID mice were injected PBL isolated from diluted (1:2) blood.
Ito et al. [87]	NOD/scid/γ^null^_c_ mouse model	NOD/Shi-scid C57BL/6J-γc null	Female NOD/Shi-scid mice were crossed with male C57BL/6J-γ cnull mice, F1 females were mated with NOD/Shi-SCID males. Males obtained were backcrossed 7 times with NOD/Shi-SCID mice. Mice obtained by 8 backcrossings were intercrossed to obtain mice homologous for the SCID and γ c null genes.
Traggiai et al. [90]	Cord blood cell–transplanted mice	Rag2-/-γc-/c-	We transplanted newborn Rag2-/- γc-/c- mice CD34 cord blood cells. Mice were subsequently analyzed between weeks 4 and 26 of age, until human CD45 hematopoietic cells were detected in all animals.
Khodayari et al. [91]	implantation mouse models	BALB/c	After were radiated by Cobalt-60 (4 Gy) 24 h the female BALB/c mice (6–8 weeks) have subcutaneously received 3 × 10^6^ MCF-7 cells in the right flank.
Yao et al.[94]	implantation mouse models	BALB/c	1. HepG2 transplantation tumor model: HepG2 cells (2 × 106) were subcutaneously injected into each mouse. Tumor-bearing mice were grouped according to the tumor volume after one week. 2. PDX model: All fragments from one hepatoma patient were subcutaneously inoculated into one flank of the experimental 5-week-old nude mice. Tumor growth was measured twice weekly using a Vernier caliper. The established PDX model was called passage 1 (P1). When the tumor size of P1 reached approximately 750 mm ^3^, the tumor was separated and sliced into small fragments (approximately 3 × 3 × 3 mm^3^/fragment) and reinoculated into mice to obtain the subsequent passages P2, P3, P4, and so on.
Su et al.[95]	Caudal vein injection mouse model and implantation mouse models	BALB/c NOD/SCID	1. HepG2 and MHCC97H cells with/without YTHDF1 knockdown were exposed to sublethal heat treatment and recovered for 1 h before injected into the tail vein of NOD/SCID mice. 2. Subcutaneous tumors were first grown through inoculating HCCLM3-NC and HCCLM3-shSTIP1 cells (1 × 107 cells/ spot) at the right flank of mice. When reached 10-mm in diameter, tumors were harvested, non-necrotic tissues were cut into 1 mm^3^ pieces and implanted into the left lobe of another tumor-free mouse’s liver. The experiment was carried out four weeks after implantation. 3. After washing out of blood and unwanted tissues, the tumor blocks were cut into pieces at 1 mm × 1 mm × 1 mm under sterilized condition. Mice were anesthetized. And a 1 cm subcutaneous pocket was made on the right flank to store the tumor piece. About three months later, successful subcutaneous xenograft was visible and could be stably passed from one mouse to another. We anesthetized the tumor-bearing mice again to harvest the tumors and cut them into pieces at 1 mm×1 mm×1 mm again. Another batch of mice was anesthetized. Subcostal incision was performed to expose liver lobes. Tumor pieces were placed into the liver via a tunnel made by microscopic forceps. Mice were resumed feeding to nourish orthotopic tumor. Successful orthotopic tumor could be palpated within 2 months.
Xun et al. [96]	implantation mouse models	NOD/SCID BALB/c	1. Fresh tumor tissues derived from HCC patients were inoculated into the back subcutaneous of 6-8-week-old NSG mice, then generated the PDX model. 2. HepG2 cells were transplanted into the dorsal flanking of 6-8-week-old male BALB/c nude mice. Then, tumor metastases to mouse liver were selected for the experiments.
Hu et al. [97]	implantation mouse models	BALB/c	Fresh HCC tissues from clinical surgical specimens were cut to a depth of 2 mm in diameter and subcutaneously buried in the right axilla of eighty nude mice by a trocar puncture. The mice were continuously fed, and the growth of the tumors was regularly observed. The tumor xenograft model was observed for 35 days. the tumors in the control group exceeded the criteria (3000 mm^3^) defined by the experimental animal ethics committee, the observation was terminated.
Nazzal et al. [98]	implantation mouse models	NOD/SCID	HCC liver specimens from HCC patient were cut into small pieces (1–3 mm^3^) and directly implanted into NSG mice. Mice were anesthetized using isoflurane (1–3%), skin aseptically prepared, and a small dorsal midline incision (< 10 mm) was made at the level of the flank. Tumor tissues were placed in bilateral subcutaneous pockets and the incision was closed. Lidocaine was infiltrated at the wound edges to control postoperative pain. When tumor volume reached > 600 mm^3^, the mouse was humanely sacrificed and the tumors were cryopreserved or explanted for passage in another NSG mouse using the same protocol. Xenograft tumor was developed after 6–7 weeks from mice and was successfully passaged in mice for three generations.

Note: HCC, hepatocellular carcinoma; DEN, diethylnitrosamine; AFB1, Aflatoxin B1.PBL; peripheral blood lymphocytes; PDX, patient-derived xenografts; NSG, NOD/SCID γ

## Humanized mice and HCC immunotherapy

Humanized, or human-like refers to the transplantation of human cells, tumor, gene, and even functional immune system into a mouse to better mimic the human tumor microenvironment (TME) and human immune system (HIS).

The model replaces the mouse liver with a human liver to create a human liver microenvironment in mice. NOG TK Tg mouse based on NOG background was established to reconstitute the human liver and showed a high level of synthetic human liver function, including expression of the liver-specific enzyme, mature human liver gene expression profile, and even human-specific drug metabolism patterns. Herpes simplex virus type 1 thymidine kinase (HSVtk) transgene was transduced into the liver of NOG mouse and then accept a brief exposure to a non-toxic dose of ganciclovir, the function of this humanized liver could be stably maintained for up to 8 months in NOG TK Tg mouse [99]. Moreover, hepatic injury caused by overexpression of urokinase-type plasminogen activator or genetic knockout of fumarylacetoacetate hydrolase gene coupled with the immunodeficient background allowed reconstitution of human hepatocytes in mice [100, 101]. These human-liver mouse models provide multiple approaches to studying pharma-cometabolism and pharmacokinetics after hepatic engraftment and the differentiation of liver stem cells.

**Fig. 2:**
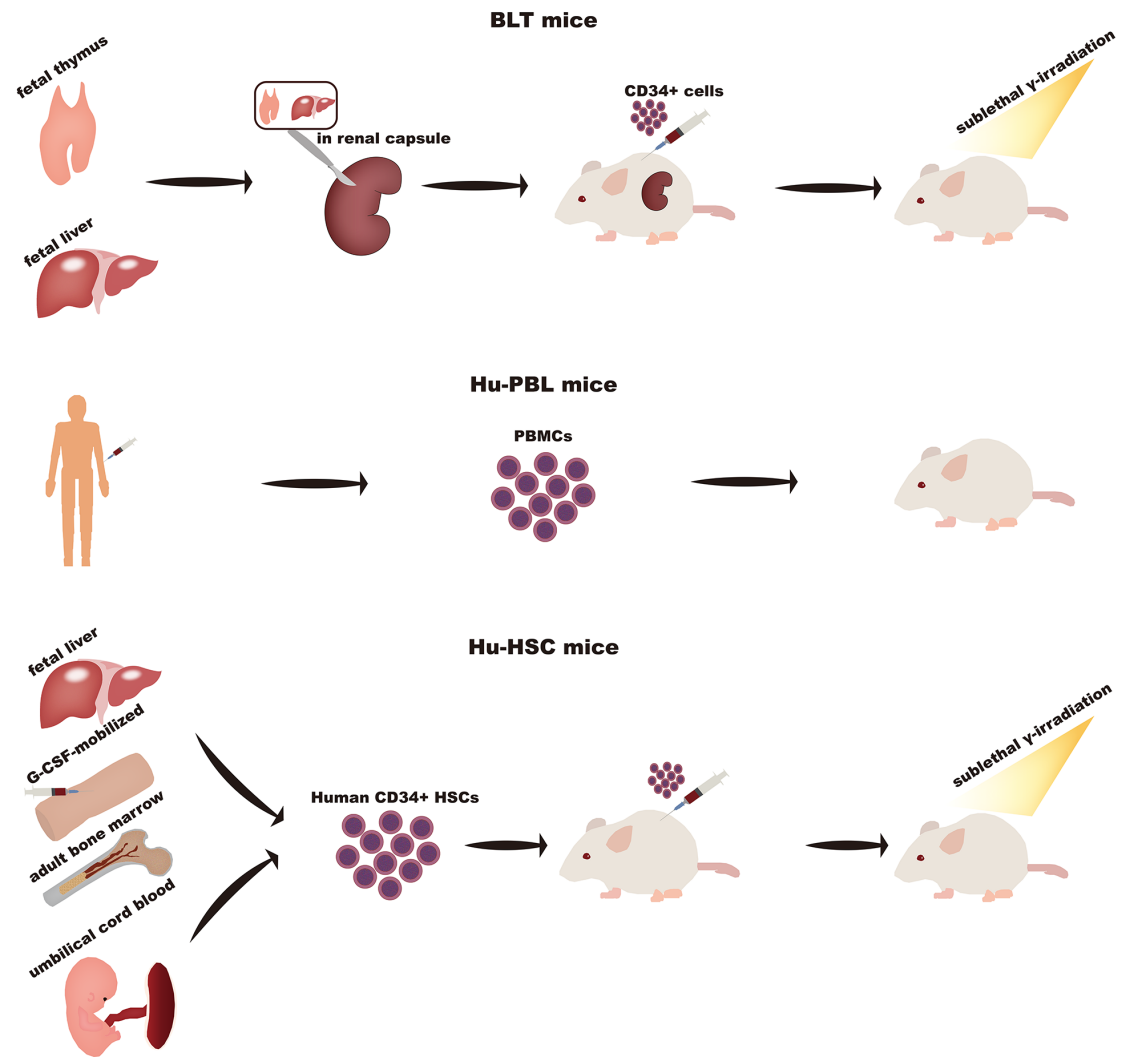
Three widely used HIS mice including Hu-PBL mice, Hu-HSC mice (or Hu-SRC, Hu-CD34 + Model), and BLT mice

For the study of immunotherapy, mice that recapitulate the human immune response are urgently required. There are three widely used HIS mice: Hu-PBL (human peripheral lymphocytes) mice, Hu-HSC (human CD34 + cord blood hematopoietic stem cells) mice (or Hu-SRC, Hu-CD34 + Model), and BLT (bone marrow-liver-thymus) mice. (Fig. 2) The Hu-PBL mice are first created by transplanting peripheral blood monocytes into immunodeficient mice, which are mainly used in viral infection (such as human immune deficiency virus or Epstein-Barr virus) or graft-versus-host (GVH) studies [87, 102]. However, the main drawback of this mouse model is its short lifespan of about 4–6 weeks [90, 103]. That means the therapeutic observational time is limited and the long-term effect of drugs doesn’t evaluate. Human CD34 + HSCs derived from human umbilical cord blood [103, 104], adult bone marrow, fetal liver [88], or granulocyte colony-stimulating factor-mobilized HSCs [105] are injected into mice followed by sublethal γ-irradiation (or busulfan, and antibody-mediated deletion) [106, 107] to eliminate mouse HSCs to facilitate human HSCs engraftment. This model has long-term engraftment and is successfully used to develop a xenotransplantation model. The BLT model is generated by transplanting human fetal thymus/liver tissues into the renal capsules of mice followed by the immediate injection of CD34 + hematopoietic/progenitor cells and then sublethal whole-body irradiation within 3 days [108]. The reconstituted human hematopoietic lineages, B cells, T cells, monocyte/macrophages, and even dendritic cells can be detected by 8 weeks. However, human MHC-restricted T-cell responses and a higher incidence of GVH occur in the Hu-BLT model [109, 110].

NSG mice, Hu-HSC mice, and hCD45-depleted humanized mice that are treated with anti-human cluster of differentiation 45 (anti-hCD45) Ab to remove human immunity were engraftment HCC patient-derived xenograft tumor tissue [83]. Compared with NSG mice and hCD45-depleted humanized mice, tumor growth is faster in Hu-HSC mice, and similarly, the proliferation and angiogenic capacity of HCC tumors are increased in Hu-HSC mice, suggesting that HCC cells may alter TME and immune system to promote tumor growth [111]. Further studies demonstrated that human immune cells, particularly intratumor hCD14 + immune cells, could be altered by HCC tumors to augment their growth in a positive feedback manner [111–115]. These findings indicate a complex interaction between TME and HCC, and it appears necessary to pay attention to the TME and immune infiltration upon discussion of the pathology and immunotherapy of HCC. In addition, the efficacy of immunotherapy drugs, namely STAT3 inhibitor C188-9, VEGF inhibitor bevacizumab, and PD-1 antibody, pembrolizumab, was also tested in this HIS humanized model. The results showed that the combination of three drugs was the most effective [111]. Similarly, pembrolizumab and CTLA-4 inhibitor, ipilimumab, were tested in another experiment [2]. Both pembrolizumab and ipilimumab were demonstrated to reduce tumor size although ipilimumab caused multiple side effects including massive immune infiltration and multi-organ dysfunction [2]. As summarized above, the HIS humanized mouse model is more optimal for characterizing tumor growth, identifying potential targets from tumors and the immune system, and predicting the therapeutic and side effects of immunotherapy drugs. However, though subcutaneously implanted human HCC-PDX in humanized mice showed similar outcomes to the clinical research, it was also not directly implanted into the human liver environment. Therefore, next-generation humanized mouse models are still required to be developed that could fully mimic the growth of HCC in a human liver environment. Combining human liver chimeric mice with HIS humanized mice may be a promising attempt. Here, we summarize all humanized mouse models of HCC in Table 5 and all the treatment options for HCC in Table 6.

**Table 5:** Humanized mouse models of hepatocellular carcinoma

Author	Mouse model type	Mouse strains	Method
Rhim et al. [99]	Hepatocyte transplantation model	EL-myc or MT-lacZ transgenic mice	10^4^ donor cells isolated from the livers of 6 to 8-week-old adult EL-myc or MT-lacZ transgenic mice were injected into the spleens of 5-11-day-old Alb-uPA recipient mice. Four to six weeks after transplantation, the recipient mice were euthanized by cadmium injection.
Azuma H et al. [100]	Hepatocyte transplantation model	Fah–/–/Rag2–/–/Il2rg–/– (FRG) mice	The mice were maintained with drinking water containing NTBC at a concentration of 16 mg/L. The hepatocytes were then transplanted into the recipient mice intraperitoneally after intravenous injection of 5 × 10^9^ units adenoviral vectors expressing human uPA. After transplantation, the concentration of NTBC was gradually reduced (1.6 mg/L on days 0–2, 0.8 mg/L on days 3–4, and 0.4 mg/L on days 5–6), and completely withdrawn one week after transplantation.
Zhou et al. [101]	Liver cancer, lymphocyte transplantation model	NOD/SCID mice	Mice were injected subcutaneously in the armpit with 1 × 10^7^ HepG2 cells which were mixed with equal volumes of Matrigel matrix. After 14 days of subcutaneous injection of HepG2 cells, the mice with grafted tumors were injected intraperitoneally with 2 × 10^7^ human peripheral blood lymphocytes from healthy populations. After 4 weeks, all mice were sacrificed.
Ito et al. [87], Bhargavan et al. [102]	PBL transplantation model	NOD/SCID mice	The mice (4 to 6 weeks old males) were engrafted by intra-peritoneal (i.p.) injection of human PBL (30 × 10^6^ cells/mouse). For infection, a single dose of 10^4^ tissue culture infectious doses-50 (100 µl) of HIV-1ADA was intraperitoneally injected (i.p.) into animals. Animals were sacrificed after 3 weeks.
Traggiai et al. [90], King et al. [103]	PMBC transplantation model	NOD/SCID mice	Mice were irradiated with 2 Gy 4 h prior to intravenous injection of varying doses of PBMC (5–20 × 10^6^ cells). After transplantation, euthanasia was performed when xenogeneic GVHD-like symptoms occurred.
Shultz et al. [88], Holyoake et al. [104]	Bone marrow, hepatocyte transplantation model	NOD/SCID mice	Mice, aged 6 to 8 weeks, were sublethally irradiated with 350 cGy from a 137Cs source 24 h before receiving an intravenous injection of human bone marrow cells or liver cells. Additionally, mice received six consecutive intraperitoneal injections of human growth factors over a 2-week period before sacrifice.
Hayakawa et al. [105]	Cord blood transplantation model	NOD/SCID mice	Conditioning of Male NOD/SCID were 7–10 weeks old mice for Transplantation. Doses of 10 and 25 mg/kg of busulfan were injected 24 h before infusion of human cells. Donor human CB cells were suspended in PBS to final volume of 500 uL and infused intravenously via tail vein.
Czechowicz et al. [106] Lan et al. [107]	Thymus, liver transplantation model	NOD/SCID mice	The mice, aged 6 to 10 weeks, were subjected to sublethal whole-body irradiation (2–3 Gy) as a preconditioning step. Within 3 days after irradiation, approximately 1 mm^3^ fragments of fetal thymus and liver were implanted under the recipient mice’s kidney capsule. After 6 weeks of human tissue transplantation, split thickness (2.2 mm) porcine skin samples were grafted onto the lateral thoracic wall of the mice. The skin grafts were assessed daily from day 7 onward up to 4 weeks, and thereafter, assessment was conducted every 3 days. Graft rejection was defined as less than 10% of the graft remaining viable.
Melkus et al. [108]	Thymus, liver transplantation model	NOD/SCID mice	6 to 8-week-old mice were anesthetized and surgically implanted with human fetal thymus and liver tissues under the kidney capsule. Three weeks after implantation, we subjected the mice to irradiation (325 cGy) from a 137Cs gamma radiation source. Subsequently, the mice were euthanized.

Note: NTBC, 2-(2-nitro-4-trifluoromethylbenzoyl)-1,3 cyclohexanedione; uPA, urokinase; PBL, peripheral blood lymphocytes; PMBC, peripheral blood mononuclear cells; GVHD, graft-versus-host disease; CB, Cord blood;

## Modeling other liver diseases and liver fibrosis in mice

HCC usually occurs in the context of chronic liver disease. Previous therapeutic strategies for advanced hepatocellular carcinoma are limited, and one-size-fits-all treatment for hepatocellular carcinoma is adopted rather than stratifying treatment according to etiology. With the increase in therapeutic strategies, systemic therapy based on the characteristics of the tumor or microenvironment is significant because of differences in tumor biology and the composition of the tumor immune microenvironment of HCCs that develop from different liver diseases [116–118]. It is recommended that the etiology should be considered when modeling HCC.

### NAFLD

The diet-induced animal model of NAFLD (DIAMOND) is a useful tool to study the development of HCC in NAFLD because of possessing similar development to human NAFLD. Male (female mice showed a sex bias) B6/129 mice (8–12 weeks old) were fed *ad libitum* a high-fat diet, high carbohydrate diet (Western diet, WD) with 42% kcal from fat and containing 0.1% cholesterol with a high fructose-glucose solution (SW, 23.1 g/L d-fructose + 18.9 g/L d-glucose). Obesity, liver injury, dyslipidemia, and insulin resistance are observed, and a fatty liver, steatohepatitis, and advanced fibrosis are sequentially developed. HCC is finally detected in 89% of DIAMOND mice between weeks 32–52 [119–121]. Mice living in thermoneutral environments (30–32 °C) have been reported to be more susceptible to developing NAFLD, and this eliminates sex bias [121]. Compared with other dietary animal models of NAFLD, the DIAMOND mice more faithfully replicate the histological phenotype and progression of human NAFLD. Transcriptomic profile and activation of related signaling pathways also show high similarity with humans [119]. The limitation of this model is mainly attributed to the suppression of cholesterol synthesis and a higher incidence of HCC, compared with humans. Moreover, it takes a long time to develop steatohepatitis and liver fibrosis (or cirrhosis), compared with other models [120]. A simpler murine non-alcoholic steatohepatitis (NASH) model using WD and CCl_4_ that can rapidly develop fibrosis and HCC was reported in 2018 [122]. The model closely replicates histological features and the transcriptomic hallmarks of human NASH [122]. Wolf et al. [123] observed that activated intrahepatic CD8 + T cells and NKT cells promoted NASH and HCC through interactions with hepatocytes in a mouse model recapitulating key features of human metabolic syndrome by long-term feeding of a choline-deficient high-fat diet. NKT cells primarily cause steatosis via secreted LIGHT, while CD8(+) and NKT cells cooperatively induce liver damage. Hepatocellular LTβR and canonical NF-κB signaling facilitate NASH-to-HCC transition. In addition to diet-induced NAFLD models, the chemical and genetic models are also used to study NAFLD and NASH. When modeling NAFLD, a single method often cannot accurately simulate the development and characteristics of NAFLD or NASH, and the combination of two or three models may achieve better results.

### Liver fibrosis

Liver fibrosis and cirrhosis can develop from a variety of liver diseases such as NAFLD, HBV, and HCV infections described above. Here are several chemically induced cirrhosis models. CCl_4_ can be used to induce hepatic fibrosis (4 weeks of twice-weekly dosing), cirrhosis (8 weeks of twice-weekly dosing), and advanced micronodular cirrhosis (12 weeks of twice-weekly dosing). Traditional methods of CCl_4_ administration include subcutaneous, and intraperitoneal injection, or inhalation use, and the SIC3 (three-weekly dosing) method may be a reliable approach to induce cirrhosis in mice with advantages of high reproducibility, low mortality, the possibility to withdraw the offending agent at different times, and the induction of hepatic lesions that more closely mirror the human cirrhosis [124, 125]. Other hepatotoxic agents used for inducing cirrhosis include thioacetamide, 3,5-diethoxycarbonyl-1,4-dihydrocollidine, and Streptozocin. Here, we summarize all the models of other liver diseases and liver fibrosis in mice in Table 7.

**Table 6:** The possible therapeutic choices of hepatocellular carcinoma

Therapeutic choices	Indications and specific programs	
Hepatectomy	Ia	Surgical resection
	Ib	
	IIa	
	IIb	Preoperative neoadjuvant therapy, induction or conversion therapy may be considered.
	IIIa	
	IIIb	
Adjuvant therapy after hepatectomy		Interventional therapy; Immunotherapy; Chemotherapy and targeted therapy.
Liver transplantation		Transplantation criteria: Milan Criteria.
		Treatment while waiting for a donor liver.
Ablation therapy	Ia	1. Radiofrequency ablation 2. Microwave ablation 3. Cryotherapy 4. Percutaneous ethanol injection therapy
	Ib	
Hepatic arterial interventional therapy	Ia	TACE (Not suitable or refused surgical resection, Liver transplantation and ablation therapy).
	Ib	
	IIa	
	IIb	1. TACE 2. TACE + Sorafenib
	IIIa	TACE (The main portal vein of the liver is incompletely blocked, or although it is completely blocked, the compensatory collateral vessels between the hepatic artery and the portal vein are formed).
	IIIb	TACE + systemic therapy
	IV	TACE / HAIC (Liver transplantation could not or refused to be performed).
Radiotherapy		1. Small hepatocellular carcinoma is not suitable for surgery or unwilling to surgery 2. Combined with TACE treatment 3. Treatment before liver transplantation 4. Hepatic portal vein or inferior vena cava tumor thrombus 5. Patients with extrahepatic metastasis
Radionuclide immunotherapy	I	1. HCC combined with TACE treatment, and not suitable for or refused surgical resection, liver transplantation and ablation therapy. 2. Patients who were not suitable for or refused surgical resection and liver transplantation after RFA.
	II	
First-line immunotherapy, chemotherapy and targeted drug therapy for advanced liver cancer	Hepatic function ChildPugh A or B ( = < 7)	1. Sorafenib 2. Systemic chemotherapy based on oxaliplatin 3. Lenvatinib 4. Donafenib 5. Atezolizumzb + bevacizumab 6. Lenvatinib + Paporizumab or navulizumab
	Hepatic function ChildPugh B (> 7) and C	Best supportive care; Palliative treatment
Second-line immunotherapy, chemotherapy and targeted drug therapy for advanced liver cancer	Hepatic function ChildPugh A or B ( = < 7)	1. Regorafenib2. Ramucirumab (AFP > 400ng/ml) 3. Cabozantinib 4. Those who have used sorafenib in the past can consider Carrelizumab + FOLFOX4 5. These who have previously used oxaliplatin can consider Carrelizumab combined with Apatinib
	Hepatic function ChildPugh B (> 7) and C	Best supportive care; Palliative treatment

Note: TACE, transcatheter arterial chemoembolization; HAIC, hepatic arterial infusion chemotherapy; RFA, Radiofrequency ablation; AFP, Alpha fetoprotein

**Table 7:** The mouse models of other liver diseases

Author	Mouse model type	Mouse strains	Method
Santhekadur et al. [119] Tsuchida et al. [121]	NAFLD	B6/129, C57Bl6/J and S129S1/svlmJ mice	Mice (8–12 weeks old) were fed ad libitum a high-fat diet, high carbohydrate diet with 42% kcal from fat and containing 0.1% cholesterol with a high fructose-glucose solution (23.1 g/L d-fructose + 18.9 g/L d-glucose). Obesity, liver injury, dyslipidemia, and insulin resistance were observed between 32 and 52 weeks.
Constandinou et al. [122]	Liver fibrosis	No special instructions	Mice were intraperitoneally injected with a 1:7 (volume/volume) mixture of CCL_4_: olive oil every 5 days (a total of 0.125 mL/g CCL_4_ per injection) for 4 weeks to induce established fibrosis, and harvested 3 days after the final injection.
Domenicali et al. [124]	Liver fibrosis	C57BL/6NCrl mice	Mice with a body weight between 20 and 25 g were pretreated with phenobarbital (0.3 g/L) dissolved in drinking water. Twice a week, 2 milliliters of a 50% (v/v) CCL_4_ solution, containing 1.0 mL per kg of body weight of CCL_4_ dissolved in liquid paraffin oil, was injected subcutaneously on the back.

Note: NAFLD, nonalcoholic fatty Liver disease

## Conclusion

Numerous mouse models provide powerful support for the study of hepatocellular carcinoma. The ideal preclinical models for hepatocellular carcinoma do not yet exist. Choosing the right animal model can speed up the translation of preclinical results to clinical applications, which would otherwise waste mounting time and resource on fruitless research. In the future, it is essential to develop a mouse model that can mimic the tumorigenesis, subsequent progression, microenvironment, and immune system of HCCs.

## Data Availability

There is no data needed to be deposited. The datasets generated and/or analyzed during the current study are available from the corresponding author on reasonable request.
